# Pituitary macroadenoma presenting with pituitary apoplexy, acromegaly and secondary diabetes mellitus - a case report

**DOI:** 10.11604/pamj.2013.15.39.2054

**Published:** 2013-05-31

**Authors:** Hudson Kamau Nganga, Reuben Paul Lubanga

**Affiliations:** 1 University of Nairobi, Medical Officer, Outpatient department, Consolata Hospital, Nyeri, Kenya; 2 University of Nairobi, Neurosurgery department, Kenyatta National Hospital, Nairobi, Kenya

**Keywords:** Pitutary macroadenoma, acromegaly, diabetes mellitus, CT scan, MRI

## Abstract

Pituitary adenomas are associated with significant morbidity. The usual symptoms on presentation are of endocrine dysfunction and mass effects. A 31-year-old African female presented with headache, irregular menses, blurring of vision in the right eye and complete loss of vision in the left eye for 1 year. She had coarse facial features, enlarged hands and feet. Her right eye had temporal hemianopia with decreased visual acuity and her left eye had no perception of light. Investigations revealed an elevated fasting blood sugar and an elevated prolactin and growth hormone level. A CT scan and MRI done showed a hemorrhagic pituitary macroadenoma. She was put on bromocriptine, ocreotide, analgesics and insulin. Thereafter, she underwent transphenoidal surgery, where near total resection of the tumor was achieved. Patient is doing well post-operatively. This case highlights the importance of the use of a high clinical index of suspicion and radiological findings in diagnosis.

## Introduction

Pituitary adenomas are the most common tumors of the sellar region. They are associated with significant morbidity, especially if they present with pituitary apoplexy. The usual symptoms on presentation are of endocrine dysfunction and mass effects. The documented endocrine dysfunction in this case was elevated prolactin levels and growth hormone levels resulting in acromegaly. On occasion, there is development of impaired glucose tolerance or secondary diabetes mellitus as was in this case. This occurs in only about 20% of acromegaly cases.

## Patient and observation

A 31-year-old African female presented to the Accident and Emergency Department of Kenyatta National Hospital with the chief complaints of headache, irregular menses, blurring of vision in the right eye and complete loss of vision in the left eye for 1 year. She was well prior to the onset of symptoms. The headache started first followed by gradual loss of vision in the left eye which progressed to complete loss of vision in that eye. This was then followed by progressive decline of vision in the right eye. The headache and decline in vision has progressively worsened over the one year period, but she reported temporary relief when on her medication. Her medication included bromocriptine and ocreotide. She also had associated symptoms of fever and night sweats. She did not complain of any nausea or vomiting. Her last menses were 2 months ago. There was no other significant history. She does not smoke or drink alcohol and has no past medical history or family history of hypertension or diabetes mellitus.

On examination, her vital signs were within normal. Of note, were her coarse facial features, enlarged hands and feet with sausage shaped fingers. Ocular exam of the right eye showed temporal hemianopia with decreased visual acuity. Examination of the left eye showed no perception of light. There were no other findings of significance.

**Figure 1:**
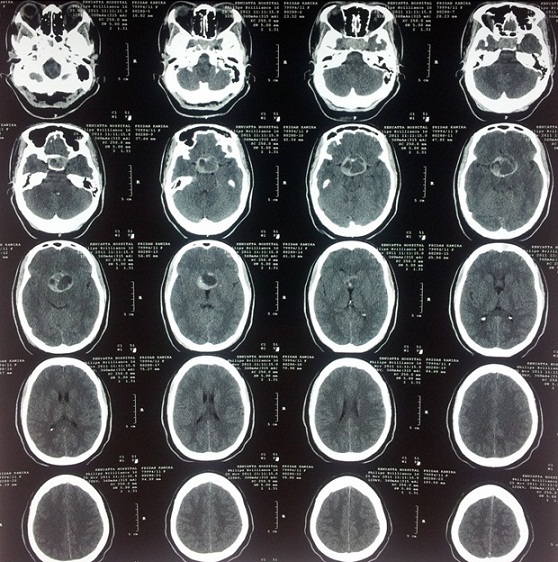
CT scan with contrast. Axial slices

**Figure 2:**
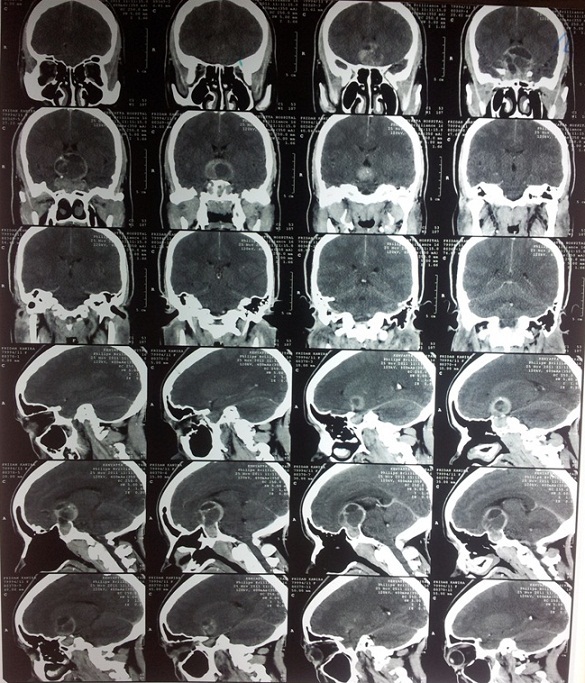
Axial slices of the patient's CT scan with contrast showing a sellar-suprasellar tumor which is cystic and has capsular enhancement

Investigations revealed an elevated prolactin level (Prolactin 1629uIU/ml), an elevated growth hormone level (Growth hormone 70.30ug/L) and an elevated fasting blood sugar (23.3mmol/L). Cortisol levels, thyroid hormone levels, thyroid stimulating hormone levels, urea, creatinine and electrolytes were all normal. HIV serology result was negative. Full blood count showed no evidence of infection. A CT scan and an MRI done both showed a suprasellar tumor with a cystic area suggestive of a hemorrhagic pituitary macroadenoma Figure 1, Figure 2.

She was put on bromocriptine, ocreotide, analgesics and insulin, and once her blood sugars were controlled, she underwent transphenoidal surgery. Intra-operatively, after opening the dura of the sella turcica, brown (motor oil like) hematoma was encountered and evacuated. Tumor was brownish purple and soft in consistency. Near total resection of the tumor was achieved. Patient is doing well post-operatively.

## Discussion

Pituitary adenomas are the most common tumors of the sellar region. They arise from epithelial pituitary cells and account for 10-15% of all intracranial tumors. Tumors exceeding 10 mm are defined as macroadenomas [1]. Pituitary macroadenomas are benign epithelial neoplasms composed of adenohypophysial cells. Primary malignant tumors of the pituitary are extremely rare. Causal contributors to pituitary tumor development include heredity, hormonal influence and genetic mutations [2].

In USA, pituitary tumors are found on autopsy in as many as 25% of unselected cases. The annual incidence of pituitary neoplasms varies from 1-7 cases per 100,000 population based on neurosurgical series. Morbidity results from mass effects, hormonal imbalance (pituitary hormone deficiency due to compression of the normal pituicytes or hormonal excess from the tumor), and patient comorbidities. Significant morbidity is also associated with treatment of these tumors. No racial predilection exists for pituitary macroadenomas and there is an equal distribution of pituitary tumors between men and women. Corticotropinomas are an exception, occurring mainly in women, with a female-to-male ratio of 4:1. Tumors affect individuals of all ages, but incidence increases with age, peaking between the third and sixth decades of life [1].

The usual symptoms on presentation are of endocrine dysfunction and mass effects but some patients may be asymptomatic. Symptoms of endocrine dysfunction depend on hormones involved. Hyperprolactinemia presents with hypogonadism, infertility, amenorrhea, and galactorrhea. Corticotropin excess presents with cushing disease and corticotropin suppression presents with glucocorticoid insufficiency. Thyrotropin excess presents with secondary hyperthyroidism and inadequate thyrotropin presents with secondary hypothyroidism [1]. Excess growth hormone presents with acromegaly, while inadequate growth hormone presents with failure to thrive in children but often no complaints in adults. About 20% of patients with acromegaly develop impaired glucose intolerance or secondary diabetes mellitus.

Gonadotropinomas most often are asymptomatic but rarely, they can lead to testicular enlargement in men and ovarian hyperstimulation in women. Deficiency of gonadotropins presents with hypogonadism and infertility. Pituitary apoplexy is characterized by sudden onset of headache, visual symptoms, altered mental status and hormone dysfunction due to acute hemorrhage or infarction of the pituitary tumor. Mass effects of the macroadenoma may present with visual deficits, headache, elevated intracranial pressure, or intracranial hemorrhage [1].

The diagnosis of a pituitary adenoma is based on clinical and radiological findings and is then confirmed by characteristic histological findings. The evaluation of a patient with a probable pituitary adenoma consists of: imaging, endocrine assessment, ophthalmology assessment and histology. On CT scan imaging, macroadenomas have variable appearances. Most are isoattenuating relative to the cortex on nonenhanced CT scans and show moderate enhancement on enhanced scans. Calcification is rare (1-8%). Necrosis, cyst formation, and hemorrhage may result in lesions of mixed attenuation. CT also shows bony changes and the mass lesion, with expansion of the sella turcica with or without erosion or depression of the sellar floor toward the sphenoid sinus. Secreting adenomas protrude or expand into the cavernous sinus more often than do nonsecreting macroadenomas [3]. In summary, macroadenomas are isoattenuating on nonenhanced CT scans and intensely enhancing on enhanced scans. On MRI, macroadenomas show a heterogeneous enhancement pattern that reflects varying degrees of necrosis, cyst formation, and hemorrhage. [4] On endocrine assessment, the hypothalamic-pituitary axis hormones, namely growth hormone, thyroid hormone, luteinising and follicle stimulating hormone should be measured together with cortisol levels and an assessment of serum and urine osmolality. It is also important to measure the random and fasting blood sugars to rule out secondary diabetes or impaired glucose tolerance. Visual acuity and visual field assessment is required to delineate any deficit. In addition, visualisation of the optic discs, to exclude papilloedema, and visual evoked potentials should be performed. On histology, pituitary macroadenomas shows varying levels of neoplastic activity. Frozen sections are usually not dependable for definitive diagnosis. Hormonal immunohistochemical stains for neuroendocrine markers are useful, especially in the nonfunctioning tumors [1, 3, 4].

The goal of treatment is complete cure. When this is not attainable, reducing tumor mass, restoring hormone function, and restoring normal vision are attempted using medications, surgery, and radiation. Pituitary macroadenomas often require surgical intervention for cure. The exceptions to this rule are the macroprolactinomas, which usually have an excellent response to medical therapy. Prolactin-secreting macroadenomas respond to dopaminergic agonists. The most frequently employed medications include bromocriptine, cabergoline, and, previously, pergolide. Quinagolide is an alternative with fewer adverse effects than bromocriptine. Prolactin-secreting macroadenomas are so responsive to medical therapy that surgery and radiation often are not used in treatment. Growth hormone-secreting tumors should be treated surgically, often followed by radiation therapy. Medical treatment is used after surgery to suppress growth hormone secretion, awaiting the occurrence of the effects of radiotherapy. Octreotide is the treatment of choice. A long-acting formulation administered monthly is now available. Somatostatin must be administered as a continuous infusion, while shorter-acting octreotide is administered tid-qid. Growth hormone receptor antagonists have been another addition to the treatment of acromegaly. Dopamine agonists also may be used but are not as effective as octreotide (approximately 30% of somatotropinomas respond) [1].

Corticotropin-secreting pituitary tumors are treated using surgery and radiation therapy (however, they are rather radioresistant). Medical therapy is reserved for patients whose therapy fails, those who decline other therapy, and those who cannot be treated otherwise. Medical therapy is divided into centrally acting agents that reduce corticotropin release and peripherally acting agents that reduce cortisol secretion or block cortisol action. Centrally acting medications (unfortunately effective in very rare occasions only) include bromocriptine, valproic acid, and cyproheptadine. Peripherally acting agents include ketoconazole, mitotane, and metyrapone. Use of such medications should be in combination with radiotherapy. Gonadotropin-secreting macroadenomas are treated surgically, followed by radiation. Medical therapy is reserved for those patients who decline definitive treatment. Bromocriptine or octreotide may be used. LH-releasing hormone antagonists may decrease hormone levels but do not affect the tumor size. Nonsecretory macroadenomas are treated surgically. If surgery is contraindicated, octreotide or bromocriptine may be tried. Thyrotropin-secreting tumors are treated surgically, followed by radiation therapy. Octreotide is quite effective in such tumors and can be used as adjuvant therapy [1].

Traditional radiotherapy using external beam radiation is used to complement surgery in inoperable cases or in patients declining surgery. The major drawbacks include delayed onset of action and high incidence of panhypopituitarism. Recent studies show the benefits of radiation [5]. Radiosurgery using a gamma knife employs a computer-assisted stereotactic mapping followed by several discrete radiation treatment fields to the tumor. This allows targeting maximal radiation to the tumor while minimizing radiation to the surrounding tissues. Incidence of hypopituitarism is less.Advances with gamma radiation are associated with a very low incidence of postradiation hypopituitarism if the radiation dose is kept at less than 15 Gy [6].

Pituitary macroadenomas often require surgical extirpation for cure. Transsphenoidal surgery is the approach of choice [7–9]. Only about 1% of patients require a transcranial approach. Compared with remission rates of 90% in microadenomas, macroadenomas with significant extrasellar extension have remission rates of 15-37% when treated with surgery alone. Radiation therapy and medical treatment often complement surgery [10]. Intraoperative MRI findings correlate with prognosis of visual deficits after transsphenoidal decompression of the anterior optic pathways. The use of intraoperative MRI may prevent revision surgery for unexpected symptomatic remnants.

In a study of 13 patients, Elhateer et al reported on the effectiveness of fractionated stereotactic radiation therapy (FSRT) in the treatment of macroadenomas. Based on an observational follow-up study (median period, 5.25 y) of 30 patients with pituitary macroadenomas (10 patients with functioning adenomas and 20 with nonfunctioning lesions) that were refractory to conventional surgical and/or medical treatment, Schalin-Jäntti et al also found FSRT to be a beneficial adjuvant therapy for these tumors.

## Conclusion

We present a case of a pituitary macroadenoma presenting with pituitary apoplexy, acromegaly and secondary diabetes mellitus. This case sparks an interesting discussion on the varied clinical presentation and management options. It also highlights the importance of the use of a high clinical index of suspicion and radiological findings in diagnosis.
